# Synthesis, crystal structure and Hirshfeld surface analysis of tetra­aqua­bis­(isonicotinamide-κ*N* ^1^)cobalt(II) succinate

**DOI:** 10.1107/S2056989018008861

**Published:** 2018-06-28

**Authors:** Sevgi Kansiz, Sergey Malinkin, Necmi Dege

**Affiliations:** a Ondokuz Mayıs University, Faculty of Arts and Sciences, Department of Physics, 55139 Samsun, Turkey; b Department of Chemistry, National Taras Shevchenko University of Kiev, 64/13 Volodymyrska Street, City of Kyiv 01601, Ukraine

**Keywords:** crystal structure, succinic acid, isonicotinamide, cobalt(II), Hirshfeld surfaces, hydrogen bonding

## Abstract

The reaction of CoCl_2_ with succinic acid and isonicotinamide in basic solution produces the title complex [Co(C_6_H_6_N_2_O)_2_(H_2_O)_4_](C_4_H_4_O_4_). The cobalt(II) ion of the complex cation and the succinate anion are each located on an inversion centre. The Co^II^ ion is octa­hedrally coordinated by four O atoms of water mol­ecules and two N atoms of isonicotinamide mol­ecules. The two ions are linked *via* O_water_—H⋯O_succinate_ hydrogen bonds, forming chains propagating along [001]. In the crystal, these hydrogen-bonded chains are linked into a three-dimensional framework by further O—H⋯O hydrogen bonds and N—H⋯O hydrogen bonds. The framework is reinforced by C—H⋯O hydrogen bonds. Hirshfeld surface analysis and two-dimensional fingerprint plots have been used to analyse the inter­molecular inter­actions present in the crystal.

## Abstract 2

In the title Co^II^ complex, both the cation and succinate anion are located about individual inversion centres. In the crystal, the ions are linked *via* O—H⋯O and N—H⋯O hydrogen bonds, forming a three-dimensional framework.

## Chemical context   

Metal carboxyl­ates have attracted intense attention because of their inter­esting framework topologies. Among metal carboxyl­ates, succinate dianions (succ) have good conformational freedom and they possess some desirable features such as being a versatile ligand because of the four electron-donor oxygen atoms they carry, and their ability to link inorganic moieties. Metal succinates are one of the best di­carboxyl­ate-based moieties that display an inter­esting structural variety. Di­carb­oxy­lic acids such as succinic acid and amides have been particularly useful in creating many supra­molecular structures between isonicotinamide and a variety of carb­oxy­lic acid mol­ecules (Vishweshwar *et al.*, 2003 ▸; Aakeröy *et al.*, 2002 ▸). Di­carb­oxy­lic acid ligands have been utilized frequently in the synthesis of various metal carboxyl­ates. For this reason they have been investigated widely, both experimentally and computationally. We describe herein the synthesis, structural features and Hirshfeld surface analysis of a new tetra­aqua­bis­(isonicotinamide-*κN^1^*)cobalt(II) succinate complex.

## Structural commentary   

**Figure 1:**
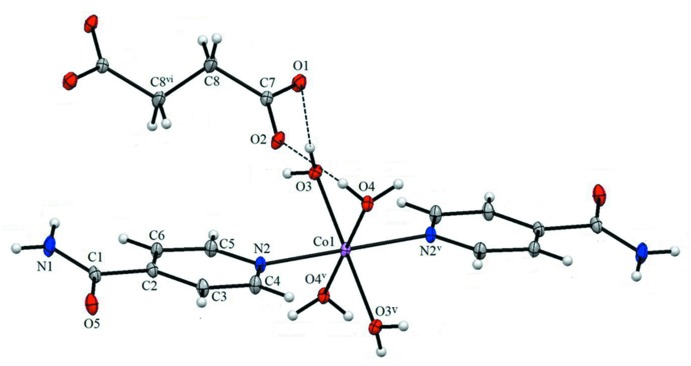
The mol­ecular structure of the title complex, with the atom labelling. Displacement ellipsoids are drawn at the 30% probability level. [Symmetry codes: (v) −*x* + 1, −*y* + 1, −*z* + 2; (vi) −*x* + 1, −*y* + 1, − *z* + 1.]

The mol­ecular structure of the title complex is illustrated in Fig. 1 ▸. The cobalt(II) ion is coordinated octa­hedrally by four O atoms of water mol­ecules and two N_pyridine_ atoms of isonicotinamide mol­ecules. The values of the Co—O_water_ and Co—N_pyridine_ bond lengths and the bond angles involving atom Co1 (Table 1 ▸) are close to those reported for similar cobalt(II) complexes (Gao *et al.*, 2006 ▸; Liu *et al.*, 2012 ▸). The C—O bond lengths in the deprotonated carb­oxy­lic groups of the succinate dianion are almost the same, *viz*. 1.247 (3) Å for C7—O1 and 1.257 (3) Å for C7—O2, indicating delocalization of charge. Each O atom of the succinate dianion is linked to an H atom of a water mol­ecule *via* O—H⋯O hydrogen bonds, so forming chains along the *c*-axis direction (Table 2 ▸ and Figs. 1 ▸ and 2 ▸).
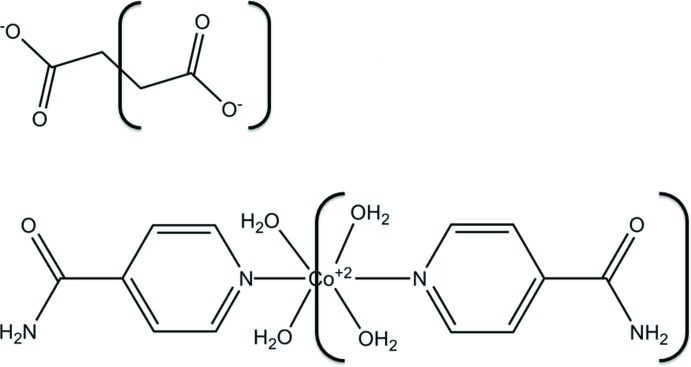


## Supra­molecular features   

**Figure 2:**
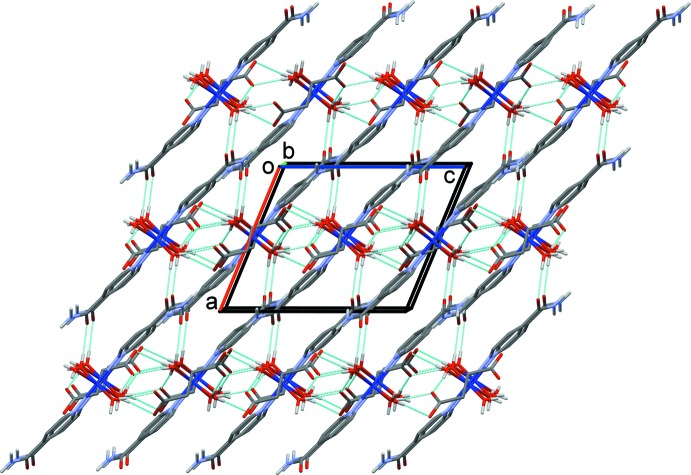
A view along the *b* axis of the crystal packing of the title complex. Dashed lines indicate the hydrogen bonds (see Table 2 ▸).

In the crystal, the chains formed by O—H⋯O hydrogen bonds involving the succinate anions and the complex cations are linked by further O—H⋯O and N—H⋯O hydrogen bonds, forming a three-dimensional supra­molecular architecture (Table 2 ▸ and Fig. 2 ▸). Within the framework, C—H⋯O hydrogen bonds are also present (Table 2 ▸).

## Hirshfeld surface analysis   

**Figure 3:**
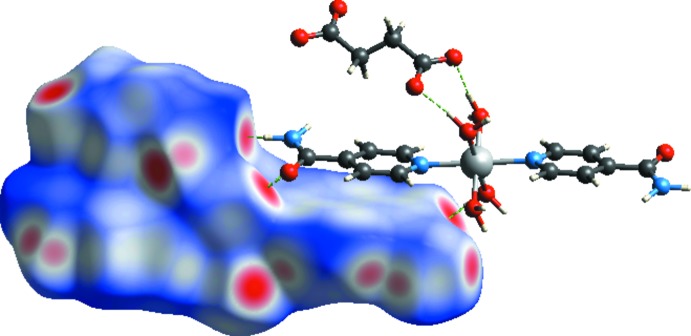
*d*_norm_ mapped on the Hirshfeld surfaces to visualize the intra­molecular and inter­molecular inter­actions of the title complex.

*CrystalExplorer17.5* (Turner *et al.*, 2017 ▸) was used to analyse the inter­actions in the crystal. The mol­ecular Hirshfeld surfaces were obtained using a standard (high) surface resolution with the three-dimensional *d*_norm_ surfaces mapped over a fixed colour scale of −0.728 (red) to 1.428 (blue). The red spots in the *d*_norm_ surface (Fig. 3 ▸), indicate the regions of donor–acceptor inter­actions given in Table 2 ▸.

**Figure 4:**
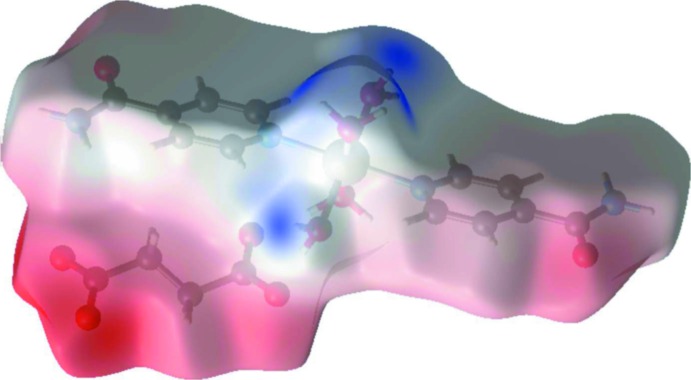
A view of the three-dimensional Hirshfeld surface of the title complex, plotted over the electrostatic potential energy.

The view of the three-dimensional Hirshfeld surface of the title compound plotted over electrostatic potential energy in the range −0.366 to 0.236 a.u. using the STO-3G basis set at the Hartree–Fock level of theory is given in Fig. 4 ▸. The C—H⋯O, N—H⋯O and O—H⋯O hydrogen-bond donors and acceptors are shown as blue and red areas around the related atoms with positive (hydrogen-bond donors) and negative (hydrogen-bond acceptors) electrostatic potentials, respectively.

**Figure 5:**
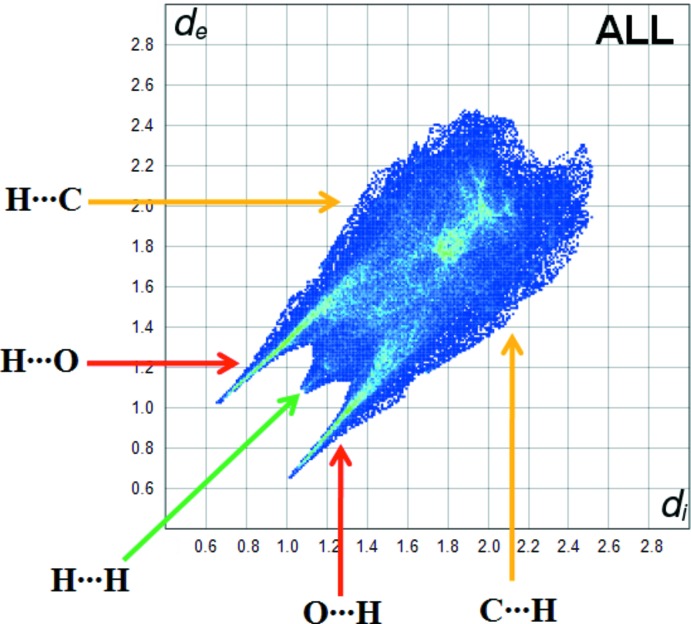
The fingerprint plot of the title compound.

**Figure 6:**
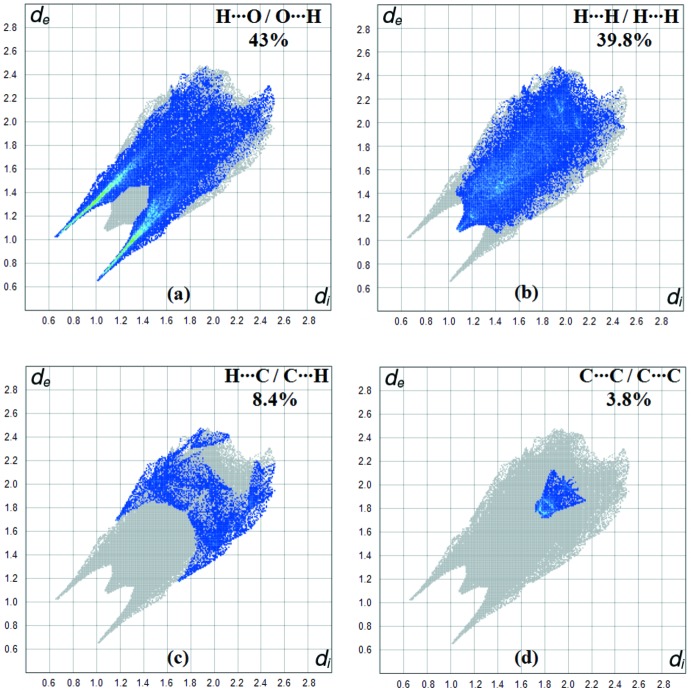
(*a*) H⋯O/O⋯H, (*b*) H⋯H/H⋯H, (*c*) H⋯C/C⋯H and (*d*) C⋯C/C⋯C contacts in the title complex, showing the percentages of contacts contributing to the total Hirshfeld surface area.

The fingerprint plot for the title complex is presented in Fig. 5 ▸. The contribution from the O⋯H/H⋯O contacts, corresponding to C—H⋯O, N—H⋯O and O—H⋯O inter­actions, is represented by a pair of sharp spikes characteristic of a strong hydrogen-bonding inter­action (43%) (Fig. 6 ▸ *a*). The H⋯H inter­actions appear in the middle of the scattered points in the two-dimensional fingerprint plots with an overall Hirshfeld surface of 39.8% (Fig. 6 ▸ *b*). The contribution of the other inter­molecular contacts to the Hirshfeld surfaces is C⋯H/H⋯C (8.4%) (Fig. 6 ▸ *c*). The C⋯C/C⋯C contacts with 3.8% contribution appear as points of low density (Fig. 6 ▸ *d*).

## Database survey   

A search of the Cambridge Structural Database (CSD, version 5.39, update May 2018; Groom *et al.*, 2016 ▸) revealed the structures of five similar tetra­aqua­bis­(isonicotinamide-κ*N* ^1^)cobalt(II) complexes with different counter-anions. They include *p*-formyl­benzoate dihydrate (HUCPIF; Hökelek *et al.*, 2009 ▸), bis­(3-hy­droxy­benzoate) tetra­hydrate (LAMMOD; Zaman *et al.*, 2012 ▸), disaccharinate sesquihydrate (LEHHUC; Uçar *et al.*, 2006 ▸), bis­(thio­phene-2,5-di­carboxyl­ate) dihydrate (NETQOU; Liu *et al.*, 2012 ▸) and terephthalate dihydrate (SETHIJ; Gao *et al.*, 2006 ▸). In all five complexes the cation possesses inversion symmetry with the cobalt ion being located on a centre of symmetry. The Co—O_water_ bond lengths vary from *ca* 2.057 to 2.115 Å, while the Co—Npyridine bond lengths vary from *ca* 2.131 to 2.169 Å. In the title complex, the cation also possesses inversion symmetry and the Co—O_water_ bond lengths [2.079 (2) and 2.113 (2) Å] and the Co—N_pyridine_ bond length [2.154 (2) Å] fall within these limits. In addition, there are several precedents for succinic acid and isonicotin­amides, including the structures of bis­(isonico­tin­amide) succinic acid (Aakeröy *et al.*, 2002 ▸), succinic acid *N,N′*-octane-1,8-diyldiisonicotinamide (Aakeröy *et al.*, 2014 ▸), succinic acid bis­(isonicotinamide) (Vishweshwar *et al.*, 2003 ▸) and *catena*-[(μ_4_-succinato)(μ_2_-succinato)bis­(μ_2_-4-pyridyl­isonicotin­amide)­dizinc] (Uebler *et al.*, 2013 ▸).

## Synthesis and crystallization   

An aqueous solution of succinic acid (25 mmol, 3 g) was added to a solution of NaOH (50 mmol, 2 g) under stirring. An aqueous solution of CoCl_2_·6H_2_O (25 mmol, 5.95 g) was added and the reaction mixture stirred for 30 min at room temperature. The pink mixture obtained was filtered and left to dry. The pink crystalline material (0.86 mmol, 0.20 g) obtained was dissolved in water and added to a aqueous solution of isonicotinamide (1.71 mmol, 0.21 g). The resulting suspension was filtered and the filtrate allowed to stand. Red prismatic crystals were obtained from the filtrate in five weeks.

## Refinement   

Crystal data, data collection and structure refinement details are summarized in Table 3 ▸. The water and NH_2_ H atoms were located from difference-Fourier maps and freely refined. The C-bound H atoms were positioned geometrically and refined using a riding model: C—H = 0.93-0.97 Å with *U*_iso_(H) = 1.2*U*_eq_(C).

## Supplementary Material

Crystal structure: contains datablock(s) I, global. DOI: 10.1107/S2056989018008861/su5446sup1.cif

Structure factors: contains datablock(s) I. DOI: 10.1107/S2056989018008861/su5446Isup2.hkl

CCDC reference: 1842712

Additional supporting information:  crystallographic information; 3D view; checkCIF report

## Figures and Tables

**Table 1:** Selected geometric parameters (Å, °)

Co1—O3	2.1134 (15)	Co1—N2	2.1540 (16)
Co1—O4	2.0795 (16)		
			
O4—Co1—N2^i^	92.15 (7)	O3—Co1—N2^i^	88.85 (6)
O4—Co1—N2	87.85 (6)	O4^i^—Co1—O3	88.82 (7)
O3—Co1—N2	91.15 (6)	O4—Co1—O3	91.18 (7)

Symmetry code: (i) 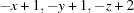.

**Table 2:** Hydrogen-bond geometry (Å, °)

*D*—H⋯*A*	*D*—H	H⋯*A*	*D*⋯*A*	*D*—H⋯*A*
O3—H3*B*⋯O1	0.79 (4)	1.97 (4)	2.756 (3)	176 (3)
O4—H4*A*⋯O2	0.77 (3)	1.88 (3)	2.651 (2)	174 (3)
O3—H3*A*⋯O2^ii^	0.81 (3)	1.92 (3)	2.729 (2)	175 (3)
O4—H4*B*⋯O5^iii^	0.83 (4)	1.97 (4)	2.801 (2)	174 (3)
N1—H1*A*⋯O5^iv^	0.92 (4)	2.34 (4)	3.227 (3)	160 (3)
N1—H1*B*⋯O1^v^	0.87 (4)	2.14 (4)	2.966 (3)	160 (3)
C5—H5⋯O2^ii^	0.93	2.41	3.307 (3)	161
C6—H6⋯O5^iv^	0.93	2.31	3.223 (3)	167

Symmetry codes: (ii) 
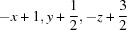
; (iii) 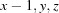; (iv) 
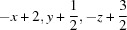
; (v) 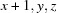.

**Table 3:** Experimental details

Crystal data	
Chemical formula	[Co(C_6_H_6_N_2_O)_2_(H_2_O)_4_](C_4_H_4_O_4_)
*M*_r_	491.32
Crystal system, space group	Monoclinic, *P*2_1_/*c*
Temperature (K)	296
*a*, *b*, *c* (Å)	9.6757 (8), 10.0381 (8), 11.4947 (10)
β (°)	112.489 (6)
*V* (Å^3^)	1031.53 (15)
*Z*	2
Radiation type	Mo *K*α
μ (mm^−1^)	0.89
Crystal size (mm)	0.68 × 0.49 × 0.37

Data collection	
Diffractometer	Stoe *IPDS* 2
Absorption correction	Integration (*X-RED32*; Stoe & Cie, 2002 ▸)
*T*_min_, *T*_max_	0.664, 0.770
No. of measured, independent and observed [*I* > 2σ(*I*)] reflections	5748, 2125, 1709
*R*_int_	0.033
(sin θ/λ)_max_ (Å^−1^)	0.628

Refinement	
*R*[*F* ^2^ > 2σ(*F* ^2^)], *wR*(*F* ^2^), *S*	0.034, 0.087, 1.02
No. of reflections	2125
No. of parameters	166
H-atom treatment	H atoms treated by a mixture of independent and constrained refinement
Δρ_max_, Δρ_min_ (e Å^−3^)	0.28, −0.38

Computer programs: *X-AREA* and *X-RED32* (Stoe & Cie, 2002 ▸), *SHELXS97* (Sheldrick, 2008 ▸), *SHELXL2017* (Sheldrick, 2015 ▸), *ORTEP-3 for Windows* (Farrugia, 2012 ▸), *Mercury* (Macrae *et al.*, 2008 ▸) and *PLATON* (Spek, 2009 ▸).

## supplementary crystallographic information

### Crystal data

**Table:**

[Co(C_6_H_6_N_2_O)_2_(H_2_O)_4_](C_4_H_4_O_4_)	*F*(000) = 510
*M**_r_* = 491.32	*D*_x_ = 1.582 Mg m^−^^3^
Monoclinic, *P*2_1_/*c*	Mo *K*α radiation, λ = 0.71073 Å
*a* = 9.6757 (8) Å	Cell parameters from 8789 reflections
*b* = 10.0381 (8) Å	θ = 3.1–30.2°
*c* = 11.4947 (10) Å	µ = 0.89 mm^−^^1^
β = 112.489 (6)°	*T* = 296 K
*V* = 1031.53 (15) Å^3^	Prism, red
*Z* = 2	0.68 × 0.49 × 0.37 mm

### Data collection

**Table:**

Stoe IPDS 2 diffractometer	2125 independent reflections
Radiation source: sealed X-ray tube, 12 x 0.4 mm long-fine focus	1709 reflections with *I* > 2σ(*I*)
Detector resolution: 6.67 pixels mm^-1^	*R*_int_ = 0.033
rotation method scans	θ_max_ = 26.5°, θ_min_ = 4.0°
Absorption correction: integration (X-RED32; Stoe & Cie, 2002)	*h* = −12→12
*T*_min_ = 0.664, *T*_max_ = 0.770	*k* = −12→12
5748 measured reflections	*l* = −14→10

### Refinement

**Table:**

Refinement on *F*^2^	Primary atom site location: structure-invariant direct methods
Least-squares matrix: full	Secondary atom site location: difference Fourier map
*R*[*F*^2^ > 2σ(*F*^2^)] = 0.034	Hydrogen site location: mixed
*wR*(*F*^2^) = 0.087	H atoms treated by a mixture of independent and constrained refinement
*S* = 1.02	*w* = 1/[σ^2^(*F*_o_^2^) + (0.0544*P*)^2^ + 0.0723*P*] where *P* = (*F*_o_^2^ + 2*F*_c_^2^)/3
2125 reflections	(Δ/σ)_max_ < 0.001
166 parameters	Δρ_max_ = 0.28 e Å^−^^3^
0 restraints	Δρ_min_ = −0.38 e Å^−^^3^

### Special details

**Table:**

Geometry. All esds (except the esd in the dihedral angle between two l.s. planes) are estimated using the full covariance matrix. The cell esds are taken into account individually in the estimation of esds in distances, angles and torsion angles; correlations between esds in cell parameters are only used when they are defined by crystal symmetry. An approximate (isotropic) treatment of cell esds is used for estimating esds involving l.s. planes.

### Fractional atomic coordinates and isotropic or equivalent isotropic displacement parameters (Å^2^)

**Table:**

	*x*	*y*	*z*	*U*_iso_*/*U*_eq_	
Co1	0.500000	0.500000	1.000000	0.02416 (13)	
O3	0.40225 (19)	0.64528 (17)	0.85930 (16)	0.0323 (3)	
O4	0.39411 (19)	0.34772 (15)	0.87503 (16)	0.0314 (3)	
O2	0.44508 (19)	0.36520 (15)	0.66478 (15)	0.0372 (4)	
O5	1.08679 (19)	0.30825 (16)	0.80841 (18)	0.0450 (4)	
O1	0.2865 (2)	0.53407 (16)	0.62211 (16)	0.0413 (4)	
N2	0.68645 (19)	0.47784 (15)	0.94294 (17)	0.0284 (4)	
N1	1.0405 (3)	0.5081 (3)	0.7144 (3)	0.0523 (6)	
C1	1.0166 (2)	0.4142 (2)	0.7847 (2)	0.0340 (5)	
C2	0.9000 (2)	0.4398 (2)	0.8388 (2)	0.0291 (4)	
C5	0.7381 (2)	0.5790 (2)	0.8958 (2)	0.0336 (5)	
H5	0.700517	0.663743	0.898389	0.040*	
C7	0.3809 (2)	0.4648 (2)	0.6004 (2)	0.0296 (4)	
C3	0.8494 (3)	0.3354 (2)	0.8895 (2)	0.0348 (5)	
H3	0.886482	0.249919	0.889455	0.042*	
C6	0.8434 (2)	0.5649 (2)	0.8438 (2)	0.0343 (5)	
H6	0.876190	0.638596	0.812443	0.041*	
C4	0.7441 (3)	0.3579 (2)	0.9399 (2)	0.0353 (5)	
H4	0.711309	0.286121	0.973646	0.042*	
C8	0.4207 (3)	0.5013 (3)	0.4888 (3)	0.0535 (7)	
H8A	0.368960	0.440472	0.420345	0.064*	
H8B	0.382876	0.590118	0.461003	0.064*	
H1A	0.982 (4)	0.584 (4)	0.697 (4)	0.083 (12)*	
H1B	1.107 (4)	0.495 (3)	0.683 (4)	0.076 (11)*	
H4A	0.403 (3)	0.355 (3)	0.812 (3)	0.035 (7)*	
H3A	0.449 (3)	0.711 (3)	0.857 (3)	0.049 (8)*	
H3B	0.373 (4)	0.612 (3)	0.792 (3)	0.055 (9)*	
H4B	0.304 (4)	0.330 (3)	0.858 (3)	0.065 (10)*	

### Atomic displacement parameters (Å^2^)

**Table:**

	*U*^11^	*U*^22^	*U*^33^	*U*^12^	*U*^13^	*U*^23^
Co1	0.0267 (2)	0.02335 (19)	0.0296 (2)	−0.00065 (15)	0.01885 (15)	0.00042 (15)
O3	0.0388 (9)	0.0293 (8)	0.0331 (9)	−0.0031 (7)	0.0184 (7)	0.0019 (7)
O4	0.0343 (9)	0.0338 (8)	0.0335 (9)	−0.0035 (6)	0.0211 (7)	−0.0022 (6)
O2	0.0519 (10)	0.0311 (7)	0.0413 (9)	0.0079 (7)	0.0319 (8)	0.0046 (6)
O5	0.0432 (9)	0.0380 (9)	0.0700 (12)	−0.0002 (7)	0.0399 (9)	−0.0086 (8)
O1	0.0469 (9)	0.0431 (9)	0.0443 (10)	0.0117 (7)	0.0291 (8)	0.0043 (7)
N2	0.0296 (8)	0.0264 (9)	0.0373 (9)	−0.0010 (6)	0.0217 (7)	−0.0014 (7)
N1	0.0506 (12)	0.0602 (14)	0.0686 (15)	0.0086 (12)	0.0479 (12)	0.0121 (12)
C1	0.0285 (11)	0.0403 (12)	0.0406 (12)	−0.0040 (9)	0.0214 (10)	−0.0073 (9)
C2	0.0257 (10)	0.0333 (10)	0.0336 (11)	−0.0011 (8)	0.0174 (9)	−0.0032 (8)
C5	0.0368 (12)	0.0252 (10)	0.0475 (13)	0.0038 (8)	0.0260 (10)	0.0045 (9)
C7	0.0323 (10)	0.0304 (10)	0.0309 (11)	−0.0036 (8)	0.0175 (9)	−0.0010 (8)
C3	0.0368 (12)	0.0258 (10)	0.0515 (13)	−0.0003 (8)	0.0276 (10)	−0.0027 (9)
C6	0.0349 (11)	0.0320 (11)	0.0450 (13)	0.0004 (9)	0.0253 (10)	0.0076 (9)
C4	0.0400 (12)	0.0262 (10)	0.0516 (14)	−0.0018 (9)	0.0307 (11)	0.0014 (9)
C8	0.0439 (14)	0.0825 (19)	0.0455 (14)	0.0135 (14)	0.0298 (12)	0.0250 (14)

### Geometric parameters (Å, º)

**Table:**

Co1—O4^i^	2.0794 (16)	N1—H1A	0.92 (4)
Co1—O3	2.1134 (15)	N1—H1B	0.87 (4)
Co1—O4	2.0795 (16)	C1—C2	1.504 (3)
Co1—N2^i^	2.1540 (16)	C2—C3	1.377 (3)
Co1—O3^i^	2.1134 (15)	C2—C6	1.381 (3)
Co1—N2	2.1540 (16)	C5—C6	1.372 (3)
O3—H3A	0.81 (3)	C5—H5	0.9300
O3—H3B	0.79 (4)	C7—C8	1.519 (3)
O4—H4A	0.77 (3)	C3—C4	1.370 (3)
O4—H4B	0.83 (4)	C3—H3	0.9300
O2—C7	1.257 (3)	C6—H6	0.9300
O5—C1	1.235 (3)	C4—H4	0.9300
O1—C7	1.247 (3)	C8—C8^ii^	1.456 (5)
N2—C4	1.333 (3)	C8—H8A	0.9700
N2—C5	1.334 (3)	C8—H8B	0.9700
N1—C1	1.317 (3)		
			
O3—Co1—O3^i^	180	O5—C1—N1	122.8 (2)
O4^i^—Co1—O4	180	O5—C1—C2	119.4 (2)
N2^i^—Co1—N2	180	N1—C1—C2	117.8 (2)
O4^i^—Co1—O3	88.82 (7)	C3—C2—C6	117.62 (18)
O4—Co1—O3	91.18 (7)	C3—C2—C1	119.32 (18)
O4^i^—Co1—O3^i^	91.18 (7)	C6—C2—C1	123.03 (19)
O4—Co1—O3^i^	88.82 (7)	N2—C5—C6	123.66 (19)
O4^i^—Co1—N2^i^	87.85 (6)	N2—C5—H5	118.2
O4—Co1—N2^i^	92.15 (7)	C6—C5—H5	118.2
O3—Co1—N2^i^	88.85 (6)	O1—C7—O2	124.09 (19)
O3^i^—Co1—N2^i^	91.15 (6)	O1—C7—C8	118.4 (2)
O4^i^—Co1—N2	92.15 (7)	O2—C7—C8	117.47 (19)
O4—Co1—N2	87.85 (6)	C4—C3—C2	119.73 (19)
O3—Co1—N2	91.15 (6)	C4—C3—H3	120.1
O3^i^—Co1—N2	88.85 (6)	C2—C3—H3	120.1
Co1—O3—H3A	120 (2)	C5—C6—C2	118.99 (19)
Co1—O3—H3B	110 (2)	C5—C6—H6	120.5
H3A—O3—H3B	108 (3)	C2—C6—H6	120.5
Co1—O4—H4A	112 (2)	N2—C4—C3	123.14 (19)
Co1—O4—H4B	121 (2)	N2—C4—H4	118.4
H4A—O4—H4B	106 (3)	C3—C4—H4	118.4
C4—N2—C5	116.83 (17)	C8^ii^—C8—C7	115.9 (3)
C4—N2—Co1	120.60 (13)	C8^ii^—C8—H8A	108.3
C5—N2—Co1	122.14 (13)	C7—C8—H8A	108.3
C1—N1—H1A	119 (3)	C8^ii^—C8—H8B	108.3
C1—N1—H1B	119 (2)	C7—C8—H8B	108.3
H1A—N1—H1B	122 (3)	H8A—C8—H8B	107.4
			
O5—C1—C2—C3	−14.5 (3)	N2—C5—C6—C2	0.2 (4)
N1—C1—C2—C3	166.3 (2)	C3—C2—C6—C5	−1.4 (3)
O5—C1—C2—C6	163.5 (2)	C1—C2—C6—C5	−179.5 (2)
N1—C1—C2—C6	−15.7 (3)	C5—N2—C4—C3	−1.2 (4)
C4—N2—C5—C6	1.1 (4)	Co1—N2—C4—C3	171.39 (19)
Co1—N2—C5—C6	−171.37 (19)	C2—C3—C4—N2	0.0 (4)
C6—C2—C3—C4	1.4 (4)	O1—C7—C8—C8^ii^	136.1 (3)
C1—C2—C3—C4	179.5 (2)	O2—C7—C8—C8^ii^	−44.5 (5)

Symmetry codes: (i) −*x*+1, −*y*+1, −*z*+2; (ii) −*x*+1, −*y*+1, −*z*+1.

### Hydrogen-bond geometry (Å, º)

**Table:**

*D*—H···*A*	*D*—H	H···*A*	*D*···*A*	*D*—H···*A*
O3—H3*B*···O1	0.79 (4)	1.97 (4)	2.756 (3)	176 (3)
O4—H4*A*···O2	0.77 (3)	1.88 (3)	2.651 (2)	174 (3)
O3—H3*A*···O2^iii^	0.81 (3)	1.92 (3)	2.729 (2)	175 (3)
O4—H4*B*···O5^iv^	0.83 (4)	1.97 (4)	2.801 (2)	174 (3)
N1—H1*A*···O5^v^	0.92 (4)	2.34 (4)	3.227 (3)	160 (3)
N1—H1*B*···O1^vi^	0.87 (4)	2.14 (4)	2.966 (3)	160 (3)
C5—H5···O2^iii^	0.93	2.41	3.307 (3)	161
C6—H6···O5^v^	0.93	2.31	3.223 (3)	167

Symmetry codes: (iii) −*x*+1, *y*+1/2, −*z*+3/2; (iv) *x*−1, *y*, *z*; (v) −*x*+2, *y*+1/2, −*z*+3/2; (vi) *x*+1, *y*, *z*.
